# Understanding student engagement in vaccination education: an interview-based multi-stakeholder study

**DOI:** 10.3389/fpubh.2024.1485498

**Published:** 2024-12-06

**Authors:** Melissa Schlopsna, Annette Scheersoi

**Affiliations:** Biology Education, University of Bonn, Bonn, Germany

**Keywords:** vaccine education, interest, students’ perspectives, vaccination experts, socio-scientific issue

## Abstract

The COVID-19 pandemic has underscored the importance of informed decision-making, especially concerning vaccination for disease prevention. This highlights the need for scientific literacy, trust, and understanding of relevant concepts such as pathogens, immune responses, and transmission pathways. Additionally, societal and ethical considerations are integral for a comprehensive approach. While collaborating with medical professionals and fostering argumentation and decision-making skills hold promise for enhancing engagement with these topics in educational settings, understanding students’ perspectives is essential for maintaining their motivation to learn and their interest in such complex subjects. Therefore, a qualitative study involving interviews with secondary school students, experienced educators, and vaccination experts familiar with school environments was conducted to identify factors fostering student engagement and interest in immunobiology and vaccines. The findings highlight focal areas of student interest in the topic and the value of involving students in lesson planning. They also underscore the importance of real-world relevance and the need for clear, student-centered communication with medical professionals. Recommendations for educators include integrating interactive learning activities, real-world examples, and case studies.

## 1 Introduction

The rapid spread of infectious diseases such as COVID-19 has underscored the critical importance of vaccinations in protecting global health. Vaccinations are widely recognized as one of the most effective tools in combating severe diseases, yet vaccine hesitancy and the global decline in vaccination rates are listed among the top 10 threats to public health (1).

Research highlights the positive impact of comprehensive public education on vaccination (2). While public education initiatives can take place in various settings, a qualitative study by Schott et al. (3) on increasing HPV vaccination rates suggests that school-based vaccine education is the most effective method for reaching a broad audience of students and their parents. This effectiveness is partly attributed to the significant amount of time students spend in school and the instructional expertise of trained educators (4).

When addressing controversial topics such as vaccination, it is crucial that school instruction extends beyond merely presenting facts. Effective education should empower students to contextualize and critically evaluate the information they receive (4, 5).

Interest in a topic is a key factor in determining the depth of student engagement (6, 7), which is particularly relevant for complex topics such as vaccination. Despite the importance of this factor, there is a significant gap in the research regarding how to foster student interest in vaccination-related topics and how to design effective, student-oriented instructional strategies (2, 4).

To address this gap, this study employs qualitative interviews and qualitative content analysis to explore the aspects of vaccine education that promote student interest. The primary focus of this study is to identify which aspects of the topic of vaccination students find particularly interesting. Additionally, it explores their attitudes toward addressing this complex topic in the classroom and their preferences regarding specific teaching methods. In addition to student perspectives, the study also gathers insights from experienced teachers and medical experts who regularly engage in vaccine education within schools.

The results of this study will not only contribute to improving vaccine education in schools but will also be used to inform an international open schooling project where schools collaborate with science experts. This project aims to create partnerships that enhance students’ learning experiences and further engage them in complex topics like vaccination.^1^

By analyzing the data from these interviews, this study aims to develop concrete recommendations for designing lessons on vaccination that enhance student interest and engagement. The findings are expected to provide valuable insights for educators, policymakers, and public health initiatives, aiming to improve vaccine education in schools as well as support innovative educational projects that foster contemporary science education.

## 2 Background

### 2.1 Interest and learning

As with all other topics, effective teaching on the theme of vaccination benefits from a teacher understanding the students’ perspectives and interests and using these insights to shape their instruction (8, 9). Several studies have shown that interest in a topic significantly influences attention and conceptual understanding (10, 11). Interest fosters positive emotions, persistence, and voluntary engagement with learning content, making learning more meaningful and effective (12, 13). Thus, promoting interest—with its emotional, cognitive, and value-related components—is a key responsibility of educational institutions (14). As a result, interest as a motivational construct and its development across learning contexts have become central themes in educational research (15).

Studies in both formal and informal learning contexts have identified numerous factors influencing interest development. These include the satisfaction of basic psychological needs—competence, autonomy, and social relatedness (16–19). Novelty is another influential factor, where encounters with new or unfamiliar situations, especially those involving discrepancy and surprise, promote interest (15, 20). Learning environments that support problem-oriented or inquiry-based approaches also foster interest, particularly when they involve physical and cognitive engagement (21–25).

Moreover, the inherent characteristics of learning topics themselves influence interest. For example, astrophysics tends to attract more interest among young people than geology, while biology topics related to zoology or human biology are generally more engaging than botanical ones (26).

Finally, individual psychological traits such as gender, prior knowledge, and self-efficacy differentially impact interest development and should be considered when designing learning environments (27, 28).

This study focuses on how to stimulate interest in vaccination and maintain it during the exploration of the topic to encourage a thorough engagement with the complex subject matter.

### 2.2 The topic of vaccination in school education

In school education, the topic of vaccination is often treated as a secondary subject, merely connected to broader topics such as the immune system or genetics (4). For example, the results of a textbook analysis in Germany show that vaccination is only a small part of the topic of the immune system and is not treated as an independent subject (29). The textbooks analyzed contain a limited selection of teaching materials, and the presentation of information usually follows a uniform pattern: details on the specific and innate immune systems are provided, followed by a chapter on active and passive immunization. This chapter is divided into therapeutic and preventive vaccination and includes information on the general effects of vaccination in the human body and the concept of herd immunity. Most books also cover Jenner’s historical cowpox experiment for the development of a smallpox vaccine and provide an example of a modern vaccine, such as for measles or tick-borne encephalitis (TBE). The tasks provided are mostly of a reproductive nature, focusing on describing, explaining, or comparing content. Evaluation, assessment, and justification are rarely included in the tasks (29). This leads to a rather superficial treatment of the topic of vaccination, raising questions about whether such an approach, which overlooks emotional and sociocultural issues, is contemporary for science education (30). If teachers want to address the topic of vaccination more thoroughly in the classroom, they must rely on external materials, which can be difficult to find and may vary in quality.

The design of the lesson and the presentation of the subject also heavily depend on the teacher’s attitudes and prior knowledge about vaccination. If the teacher is critical of the topic, this will be reflected in their teaching [cf., (31)].

Another barrier is the lack of inclusion in university teacher education—at least in Germany; vaccination education is not part of the teacher training curriculum. Additionally, school conditions, such as the minimal integration of the topic in the core curriculum, pose challenges. The narrow guidelines of the core curriculum often leave no time for a more detailed exploration of the topic. To effectively integrate and address vaccination in the curriculum, it must be firmly incorporated into teacher education and expanded in school core curricula (2).

Given that vaccination is a complex and controversial topic, a sensitive approach to the content is also important in school education (9). Individuals who are critical of the topic are often stigmatized and labeled as poorly informed or selfish. Since students’ motivation to engage with a topic also depends on whether they feel socially included, it is crucial to create an atmosphere where even those who are critical of the topic feel respected and taken seriously. This means addressing any concerns and fears associated with this criticism (4).

To foster students’ understanding of the “Nature of Science” [e.g., (32, 33)], the topic of vaccination is particularly suitable, as historical case studies or discussions about the effectiveness and use of different vaccines allow students to critically engage with research (4). Sociocultural and ethical questions can also be integrated (e.g., vaccine scarcity and distribution or vaccine mandates) and discussed through role-playing or debates—either as part of science education or through an interdisciplinary approach in the context of geography, history, or philosophy (4).

Comprehensive engagement with the topic of vaccination and examination from a systemic perspective can also be achieved through its inclusion in One Health education, which explicitly focuses on promoting critical thinking (34).

To specifically address students’ concerns and uncertainties, schools can invite external experts, such as vaccination doctors [cf., (2)]. They can visit individual classes and answer students’ questions. This also includes expertise on current misinformation and conspiracy theories circulating on social media about certain vaccines, such as infertility claims related to COVID-19 vaccines. Misinformation can thus be specifically debunked, and students can be factually informed. In addition to their specialized expertise, the openness of students and the lower level of embarrassment when interacting with individuals outside the school context also support the inclusion of experts (2).

Few studies have explored how to generate interest in vaccination among learners or provided concrete recommendations for designing engaging lessons on this topic [cf., (4)]. This study aims to fill that gap by identifying factors that enhance students’ interest in vaccination and offering guidelines for creating lessons that effectively foster this interest.

## 3 Materials and methods

### 3.1 Data collection

To gather students’ perspectives and measure their interest, both quantitative and qualitative methods can be employed (14). For this study, qualitative data collection was chosen in the form of semi-structured interviews, as it provides the most direct form of interaction with the participants, allowing the interviewer to respond directly to statements. This approach enables follow-up questions and allows participants to elaborate on and explain aspects in detail when necessary (35). Additionally, interview participants can introduce additional aspects that the interviewer can then address with spontaneous questions and prompts (36). The interviews with the students were conducted in small groups (2–4 students) to reduce anxiety and create an atmosphere of casual conversation among peers, where they could freely exchange ideas and express their preferences and interests. This approach typically results in more authentic responses, and the interviewer needs to intervene less, thereby reducing the risk of unintentionally influencing the participants (36).

Niebert and Gropengießer (36) provide recommendations for designing such interviews with students to obtain comprehensive data. These recommendations were implemented in the context of the interviews with the students in the following manner:

To ease the atmosphere and initiate conversation, the interview began with an open prompt: “When you think about your time in school, what have you enjoyed most in biology class so far?”

To illustrate and support the discussion, to encourage the flow of conversation, and to guide their responses, participants were provided with *material*—topic sheets and custom-made info cards on various vaccines were used.

Additionally, targeted *follow-up interventions* were employed, such as asking further questions to obtain detailed information and more comprehensive explanations, e.g., “Earlier you mentioned XY, could you explain what you meant by that?”

*Ad-hoc interventions*, in the form of spontaneously generated follow-up questions, prompts, and remarks from the interviewer, were intentionally used to make the participants’ statements understandable and clear.

To ensure that no important aspects from the participants’ perspectives were overlooked during the interview, they were asked at the end if they had any additional tips or comments or if something relevant to them had been omitted (*final intervention*).

**Figure 1:**
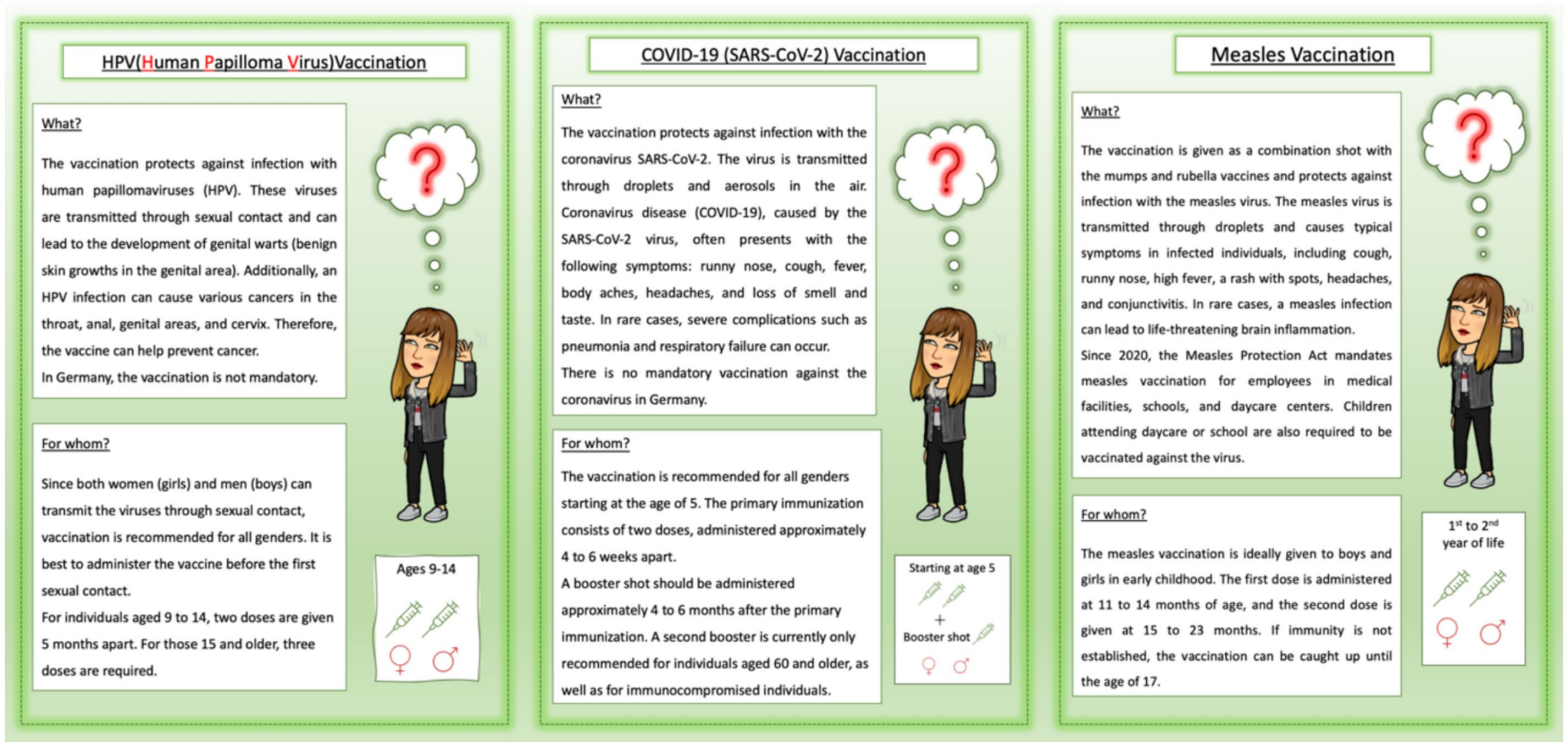
Information sheets on viruses and vaccinations (M. Schlopsna; translation from the German original).

The interview questions initially focused on the students’ general assessment of the topic of vaccination in biology class (“Would you find this topic interesting? Would you like to study this in class?”) and on their interest for vaccines against specific viruses (measles, COVID, HPV—“Which of these topics interests you the most? Which would you like to cover in class?”). Prior knowledge was briefly addressed (“Do you already know a little about the different vaccinations or viruses?”) to then use the information material to establish a common foundation for the rest of the interview (“Read through the fact sheets at your own pace to get an idea of what it’s about.”). The materials consisted of custom-made, short, and colorful information sheets on the three viruses and vaccinations (Figure 1).

The next interview question addressed specific content that the students would like to explore in class (“If you were to study the topic of vaccination in class, what would interest you the most? What would you like to discuss in class?”) to identify areas of interest.

Since addressing the topic of vaccination in class can also include social and ethical questions, the students’ views on this aspect were subsequently explored (“Would you be interested in the societal aspect? Like addressing the ethical aspects of vaccination?”). Additionally, the interview specifically inquired about anti-vaccination movements and myths and how the students perceived the potential for conflict in the classroom if these topics were discussed (“Do you think it would be problematic to talk about anti-vaxxers in your class?”).

Another set of questions focused on excursions and meetings with experts, particularly reflecting on the students’ past experiences and their evaluation of those situations (e.g., “Have you ever gone on excursions and talked to experts in your science classes? Or have experts ever come to your school?”).

Finally, the students were asked to provide tips for the methodological design of lessons on vaccination, allowing their preferences to be considered (“What tips would you give us for making the topic of vaccination interesting in class? How would you like to learn about it?”).

Due to the complexity of the topic and the integration of vaccination into the school curricula from grade 9 onwards, the study focuses on the interest development of secondary school students from grade 9 and above. The sample of interview participants consists of a total of 12 students from two grade levels (14 years old, *N* = 7; 17 years old, *N* = 5). Purposive sampling was employed to select participants who could provide rich, relevant insights into the research topic. Participants were chosen based on their age, as they are at a stage where they should have learned about basic immunobiology (and vaccines) in school, and due to the relevance of the topic to their everyday lives, e.g., HPV vaccines in adolescence. Detailed information about the study’s purpose, the importance of their participation, and the potential impact of their responses was communicated to both the students and their parents. Parental consent was obtained for all participants, and students’ confidentiality was ensured throughout the process. To ensure credibility and dependability, member checking was employed by sharing key findings with participants for confirmation.

The main objective of this study was to investigate student interest and their perspective on vaccine education. Consequently, the majority of data collection was focused on students. To supplement the data, additional interviews were conducted with biology teachers (*N* = 2) who have previously taught the topic of vaccination and the immune system, as well as with medical experts (*N* = 5) from the volunteer organization “Get vaccinated!” These experts regularly visit schools to provide vaccine education. Teachers and experts served as complementary sources, providing context and a broader understanding of student responses within the framework of educational practices, observed behaviors, and classroom interactions.

The teachers were interviewed individually, while the experts were interviewed as a group due to their availability and time restrictions.

During the data collection, the method of triangulation was employed by revisiting the same aspects across different interviews—from students, teachers and experts [triangulation by data source (37)]. This approach allows for the comparison of participants’ statements, which increases the validity of interpreting similar responses. For example, teachers and experts were asked which topics, based on their experience and observation, the students find most interesting. The interviews with teachers also served to gather their experiences in teaching the subject, as well as the limitations and challenges they face—such as involving experts in the classroom. The group interview with the experts aimed to derive recommendations for designing interest-enhancing lessons on vaccination based on their experiences in classroom settings.

### 3.2 Data analysis

The interview data were recorded via audio and subsequently transcribed into a text file (38). Additionally, the transcripts were annotated with comments in square brackets to capture non-verbal cues such as facial expressions and gestures, like laughter, fascination, or disbelief (39).

The data were then subjected to qualitative content analysis, where the statements from the transcripts were condensed into thematic units and organized into categories [content-structuring qualitative content analysis according to (40)]. These categories were deductively derived from the research questions, with additional inductive categories created for emerging themes that had not been previously captured.

## 4 Results

Through data analysis, specific areas of interest and various influencing factors for the development of interest in the topic of vaccination were identified. Additionally, the respondents provided concrete suggestions for structuring lessons, including on the topic of vaccination.

To enhance transparency, the results section includes original interview quotes alongside their categorizations and interpretations (Tables 1–6). This allows readers to critically assess the data interpretation and ensures a clear, traceable link between the raw data and the conclusions. Quotes from students are marked with ‘S’, S1–S7 representing students from grade 9 (14 years old), and S8–S12 representing students from upper secondary school (17 years old). The quotes from the two teachers are marked as T1 and T2, while the expert statements are labeled E1-5.

**Table 1:** Topic-specific interest in biology class (quotes from S=Student, T = Teacher, and E = Expert; the age of the students is indicated in each case).

Theme	Subtheme	Category	Example quotes
Interest in Human Biology	Personal relevance	Understanding one’s own body	*“I think it’s overall about understanding how the body works exactly. And so that I know how I function, so to speak. Because it also has something to do with me.” (S2, 14y.)*
	Tangible content	Topics easier to visualize	*“I can imagine that it’s simply because you could picture something, and it wasn’t something like cell types or DNA, where you could not really visualize it. It was something you could actually see.” (S1, 14y.)*
Interest in vaccination	Knowledge gaps	Limited prior knowledge of vaccination	*“I think we have already covered it, but not really in-depth. And there’s not much that I can remember now.” (S9, 17y.)* *“Usually, it’s like you get vaccinated, and your parents send you there. I’ve been vaccinated many times, and that’s it. I did not necessarily know what kind of vaccine it was.” (S11, 17y.)* *“Yeah, for example, I did not know that there are different types of vaccines. I always thought they just put a little bit of the disease into your body and then the body is like, ‘Ah, there’s a disease.’ And then it’s ready, I do not know. I did not know there were different types.” (S5, 14y.)* *“And that was always a signal to me that this is something unfamiliar, even though they had heard of the disease, but maybe did not really know what it was. And then they found it really interesting to hear about it.” (E5)* *“It seems that at home, for some, it’s not a topic either. There were those who are vaccinated, who also knew about it, especially from home. But those who were not vaccinated looked at me like, ‘What’s HPV?’ And I mean, this was in 9th grade.” (T1)*
	Relevance to daily life	Vaccination affecting everyone	*“Yeah, I think I would find it quite exciting because it’s something that affects all of us, because I think everyone has been vaccinated at some point.” (S1, 14y.)* *“We all get vaccinated (…) and you never really knew what was actually happening in the body, so if you then learn about it in school, I think it would be very interesting.” (S9, 17y.)* *“And I think it’s also important because vaccinations became more relevant because of COVID. That you learn about it to see what it actually is and how it works? And why it’s not necessarily dangerous. And that’s why I think it’s good to cover it in school.” (S8, 17y.)* *“In general, I think this is always a topic that students are interested in because it directly affects them and is part of their personal environment. So, it’s not as abstract as some other topics. I always had positive feedback, and they were happy to work on this topic.” (T1)* *“I think it’s also the public fascination with this topic. It’s something that affects all of us, and everyone wants to discuss it in some way, and that’s clearly reflected in the kids’ reactions.” (E3)*

**Table 2:** Vaccine aspects of particular interest to students (quotes from S = Student and E = Expert).

Theme	Subtheme	Category	Example quotes
Interest in vaccine mechanics	Biological processes	Functioning; mode of operation	*“I think ‘How does the vaccine work in the body?’ is the most interesting (…). The first question I ask myself when I get vaccinated is: ‘What does it trigger in my body?’“(S8, 17y.)* *“I’m also interested in vaccination because you mostly just see the external part, like getting a shot in your arm, but you do not really get an explanation of what happens inside your body.” (S4, 14y.)*
		Side effects	*“Sometimes you also get sick afterwards, or have side effects, and you do not really get an explanation of why you have those side effects or not.” (S4, 14y.)*
Interest in vaccine development	Research and development	Process of vaccine development	*“I’m also interested in how a vaccine is developed. Because first, you have to figure out what it’s for. And how long it takes to make a vaccine that might be effective.” (S4, 14y.)* *“And especially with something like COVID, there still aren’t any real long-term studies. And that’s why I find it interesting how they determine whether a vaccine is worth it or not.” (S1, 14y.)*
Interest in ethical and social aspects	Misinformation and vaccine debate	Desire to understand and form own opinions	*“But because there’s a lot of debate about vaccinations (…) I think it’s important because everyone needs to form their own opinion on it.” (S12, 17y.)* *“For yourself, I think also, like how information is presented. So that you can think about it on your own.” (S11, 17y.)* *“There are so many different opinions about it on the internet. And it’s important to get information so that you can form your own opinion.” (S10, 17y.)*
Interest in disease symptoms	Symptoms and visuals	Effect of visual aids	*“When I used to give school presentations, I always noticed that when I talked about diseases and showed pictures of them, and described what they were like and what the symptoms were, the class often got really quiet. Before, there was some rustling and whispering, but then the students suddenly became very focused and quiet and listened very attentively.” (E5)* *“In our HPV topic, it was quite exciting because we also showed some images of genital warts and things like that, and that was a moment when they were like, ‘What exactly is that?’“(E2)*
Interest in the immune system	General/basic understanding	Functioning of the immune system	*“That was the series on the immune system. The substance was injected so that the body could already develop the antibodies for it. I thought it was good to learn something about the immune system because then you really developed an understanding of it.” (S1, 14y.)*

**Table 3:** Students’ interest in specific pathogens (quotes from S = Student, T = Teacher, and E = Expert).

Theme	Subtheme	Category	Example quotes
Interest in COVID-19	Topicality and daily impact	Relevance of current events and research	*“For me, COVID would definitely be included because it’s so current.” (S6, 14y.)* *“There’s still a lot of research being done on COVID, and that’s just interesting.” (S2, 14y.)* *“Afterward, we did a small unit on the current COVID vaccines because it was, of course, exciting and something they wanted to learn about.” (T1)* *“They found it very interesting. I mean, COVID made it even more relevant.” (T1)*
	Knowledge gaps	Limited knowledge on vaccine mechanics	*“You already know what happens when you get COVID, but not really what exactly happens when you get the vaccine or what exactly the vaccine does. Or what causes you to be protected afterward.” (S6, 14y.)* *“The interest was very high. The students also researched on their own and invested time at home, informing themselves about mRNA vaccines. And they also had very specific questions about it.” (T2)*
		Limited knowledge on vaccine development	*“What I never really understood was how they approach it, like how they even come up with vaccines.” (S12, 17y.)*
	Lack of need	Overexposure leading to disinterest	*“I think the Coronavirus has already been discussed so many times, so it’s kind of been played out.” (S5, 14 y.)*
Interest in HPV	Knowledge gaps	Low awareness of consequences or relevance	*“Yes, so I was at the gynecologist, and there’s a very brief conversation just before. Um, and then an appointment is made. At least that’s how it was for me. It was briefly explained that it’s a sexually transmitted disease and that it would be very good to be vaccinated against it.” (S4, 14y.)* *“We only talked about it privately. When we got vaccinated. And that it’s a sexually transmitted disease.” (S3, 14y.)* *“[fascinated] Yeah, I did not know that HPV also leads to cancer in the throat area.”(S1, 14y.)*
	Personal relevance	Increased interest after additional information	*“Now that I know what it is, I also see the relevance in it. I also generally think that this could be a topic that might be uncomfortable for some. But I still think it’s important that it continues to be de-tabooed in schools. I do not know how to say it correctly, but that topics like HPV are normalized. And since it corresponds to the age you are when you attend school, it is relevant. Personally, I did not know about the vaccine at all, and I wasn’t aware of it, but now I see that it’s actually important to know about it and be educated on it.” (S8, 17y.)*
		Age-appropriate	*“And maybe also HPV, because we are at an age where the vaccine is relevant for our protection.” (S1, 14y.)* *“Because it’s very current among teenagers.” (S5, 14y.)*
Interest in Measles	Knowledge gaps	Little knowledge about the disease’s severity and about vaccine mechanics	*“Yeah, (…) and maybe also something about measles, because I do not have a clear picture in my mind. Just kids with some red spots.” (S1, 14y.)* *“Why exactly is it mandatory, for example?” (S7, 14y.)* *“I did not realize that it could become life threatening after a certain period because it can also affect the brain. (…) I cannot really imagine how a vaccine helps with that. So I actually find that quite interesting.” (S1, 14y.)*
	Lack of personal relevance	Low interest due to perceived outdated relevance	*“So, measles, (…) I feel like it’s been a while since it was really an issue for us.” (S12, 17y.)* *“It’s just mandatory. With optional things, it’s more interesting because then you can decide for yourself. If it’s mandatory, you just have to do it anyway. And the vaccination does not really affect us much now because we got it a long time ago.” (S12, 17y.)*
Interest in other Pathogens	Personal relevance	Exotic diseases, e.g., when traveling	*“And when it comes to vaccines in general, I would also be interested in which ones (…) because we once planned to fly to Africa and before that, we had to get a lot of vaccinations, like, uh, hepatitis or something? And, um, I would be interested in which diseases or like in different countries because in Germany there aren’t that many diseases, but what different diseases are very common in other countries.” (S4, 14y.)*
	Symptoms	Impact of symptoms	*“And I think that was just a topic that really interested them. When they saw, how does yellow fever affect you. Like how dangerous it is.” (T1)*

**Table 4:** Students’ ideas about interest-promoting biology classes (quotes from S = Student).

Theme	Subtheme	Category	Example quotes
Student-centered learning	Station learning	Control over learning	*“I think for me, I always found station learning not too bad because we worked through things ourselves step by step and could set our own pace.” (S3, 14y.)*
	Group work	Social learning	*“Usually in biology class, you just get worksheets that you have to work on, and it’s usually more fun and engaging when you do group work or work at different stations and talk to each other.” (S3, 14y.)*
	Student involvement in planning	Personal relevance and purposeful learning	*“Yeah, I think it would be good to listen to the students themselves about how they want to learn instead of just strictly sticking to the teacher’s own lesson plan […]. Maybe just take suggestions and ideas from the students.” (S1, 14y.)* *“I also think in general that lessons should be designed to be practical or related to everyday life. So you can better understand what it means for yourself. That makes it more interesting.” (S7, 14y.)* *“Yeah, so you can see the purpose behind why you are learning it.” (S4, 14y.)*
Teacher-centered instruction	Teacher lecture	Low engagement	*“What’s sometimes a bit boring is when the teacher stands at the front and talks about something because you cannot necessarily follow along the whole time.” (S2, 14y.)*
		Overload	*“It’s way, way too much in class. You cannot keep up (…) I cannot keep up anymore, and then it’s not fun anymore.” (S12, 17y.)*
Visual and experimental learning	(Hands-On) Experiments	Improved understanding	*“But when you do experiments together—not anything extreme, but just things that make concepts more tangible, or sometimes even hands-on—that really helps you understand better.” (S5, 14y.)*

**Table 5:** Suggestions for designing interest-promoting lessons on vaccination (quotes from S = Student, T = Teacher, and E = Expert).

Theme	Subtheme	Category	Example quotes
Complexity of vaccination topic	Need for simplification	Reducing complexity of biological content	*“So, yeah. Definitely, especially because vaccinations are also like photosynthesis —you hear about it all the time, but you do not really understand it.” (S8, 17y.)* *“It’s a very complex topic that needs to be didactically reduced (…). So it’s not too abstract and difficult, or the students will lose motivation right away. You cannot go as deep as you might want to because it would be too difficult and also for time reasons.” (T2)*
	Appropriate for higher grades	Age-appropriate learning	*“It’s already very complex, the topic of immunobiology is not something that I think should be taught before grade 9. With all the different things, whether it’s T-cells, plasma cells,* etc.*, the whole complex.” (T1)*
Illustration, visualization	Case studies, personal stories	Making content relatable	*“I find it really exciting when you go over old cases. For example, if you now know that there was already a vaccine back then, where everyone had to be vaccinated. And that had big effects.” (S3, 14y.)*
		Relating topic to personal life	*“When you have cases and deal with people, maybe in a film, who have had experiences with it. (…) Because then you can imagine it much better and relate it to your own life. You have an example in front of you and a certain idea of it.” (S3, 14y.)*
Application of knowledge	Transfer tasks	Developing/ exploring knowledge independently	*“They did look at something about these vaccines at some point, but when they really know exactly how mRNA is created in genetics, then they can also independently deduce, and I always let them do that. So that they can independently develop ideas about how this vaccine might work and what it actually does. And they always find that quite exciting.” (T1)*
	Real-life applications	Linking different concepts	*“They found it very interesting because mRNA and genetic engineering were our topics in genetics, and they could then apply that to vaccination.” (T2)*
Creative and playful approaches	Quizzes, games	Interactive learning	*“Like maybe doing a quiz on it and finding out a bit more about how it works.” (S6, 14y.)* *“Last time we also incorporated a game with drinking cups, (…) in the end, you could see that the more contacts you have, the higher the probability that you were (…) infected, so to speak. That went over very well.” (E4)*
	Comics	Visual/Artistic learning	*“They enjoyed creating comics. I know that always sparks great interest because it’s just a nice way to apply knowledge, to visualize it in drawings.” (T1)* *“(…) that you do more creative stuff. Because many learn better through creative things and visualizations.” (S9, 17y.)*
Sensitive and factual approaches	Neutral and pluralistic presentation of content	Avoiding judgment or exclusion	*“And you might need to be careful not to pressure them too much or say, ‘You were raised wrong!’ That’s not productive either. (…) but I think continuing to educate and present it neutrally without pushing an opinion is important.” (S8, 17y.)*
		Fostering evaluation skills	*“Choosing the course content so that the students can eventually form their own fact-based opinions. You were bombarded with opinions during the COVID pandemic, and that caused a lot of uncertainty. So they should be able to form their own opinions on such topics.” (T2)*
Social and ethical aspects in Biology class	Inclusion is questioned	Preference for social studies	*“I think that does not really belong in biology. We’ve already discussed it in religion class, but I do not think it has much place in biology.” (S9, 17y.)* *“I would put the social stuff more in the politics class. That’s where you could discuss anti-vaxxers and conspiracy theorists.” (S3, 14y.)*
		Impact on time for scientific content	*“I think the discussions [about it] are very important, but I think (…) if you also spend a lot of time on such ethical debates, then (…) you cannot do something else.” (S10, 17y.)*

**Table 6:** Interest-supporting expert-student interactions (quotes from S = Student and E = Expert).

Theme	Subtheme	Category	Example quotes
Expert presentations	Presentation quality	Complex and adult-oriented	*“With many people, you also feel like they do not really know how to convey it. They have very interesting topics, but how to communicate it so that it’s interesting but not too complex, they do not always get that.” (S7, 14y.)* *“What’s important when experts come is the presentation, because that’s often not so interesting. It needs to be well-designed.” (S11, 17y.)* *“It was a bit too adult-oriented for my taste. (…) Especially the workshops, which were not really workshops but just two-hour presentations.” (S5, 14y.)*
		Use of specialized language	*“They sometimes have a different way of speaking. I do not know if they think we automatically understand it. (…) And then they thought, with that, everything was said, but they did not really consider that students or other people might not fully understand it.” (S4, 14y.)*
Expert-student interaction	Respectful interaction	Students need to be taken seriously	*“The problem wasn’t the language, but how they treated us. One of them spoke to us as if we were kindergarten kids.” (S1, 14y.)*
		Understanding and patient experts	*“They were also more understanding. You could keep up. There was time, and you could speak up without being constantly interrupted.” (S12, 17y.)* *“There were new things since we did not cover it in class, but it was okay. They always explain the technical terms and give context.” (S9, 17y.)*
	Interaction setting	Small and intimate settings preferred	*“But it wasn’t just our class there; it was much larger, and that’s a different atmosphere, let us just say, than if it was just our class and this expert.” (S5, 14y.)*
	Communication style	Perceived easier for younger experts	*“I can imagine that maybe because they do not seem as old as the teachers, and you can communicate more on the same level.” (E4)*
Perceived value of expert engagement	Accessibility of the content	Complex content is simplified	*“Yeah, it was also just a bit easier than in regular class.” (S12, 17y.)*
	Authentic and tangible insights	Real-world perspectives	*“It’s always cool when someone from outside comes in because you get a different perspective.” (S11, 17y.)* *“You also gain new insights into what everything looks like in reality.” (S11, 17y.)*
		Research process	*“That the experts might guide us through the process or illustrate how the vaccine is produced, how they find it out. I find that interesting.” (S9, 17y.)*
	Career insights	Information about careers related to the field	*“They were relatively open and often asked questions about medical studies. In biology courses, there are often students interested in medicine.” (E4)*

### 4.1 Topic-specific interest/content aspects

When asked which topic the students found most interesting in biology class so far, human biology topics were mentioned particularly often. The students described these topics as the most exciting and memorable, citing the personal connection to their own bodies as the main reason for their interest. The tangible nature of the content also contributed to the popularity of human biology.

Half of the respondents reported having no prior knowledge of vaccination, while the other half rated their knowledge as limited, despite having covered the immune system in class. This lack of knowledge was confirmed by experts and teachers. However, the interview data show that the students want to learn more about the topic, seeing its importance both on a societal level and in relation to their own lives, especially in the context of the COVID-19 pandemic. Teachers and experts also confirmed this interest, attributing it to the personal relevance and current significance of the subject.

In summary, all students expressed an interest in studying vaccination in biology class. Their positive emotions are closely linked to the topic’s strong connection to their personal lives and health.

**Figure 2:**
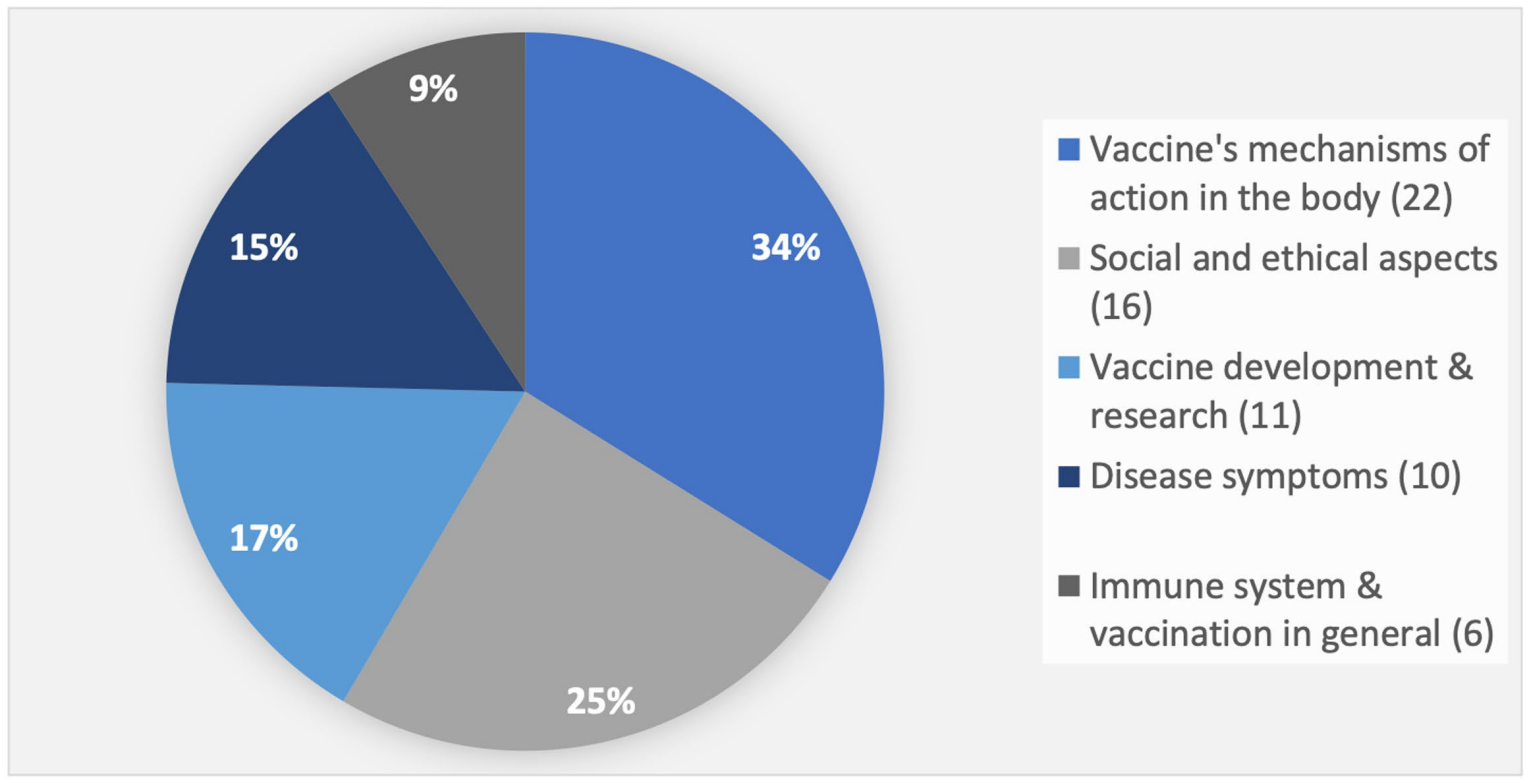
Students’ interest in different aspects of the topic of vaccination (the number of respective statements is indicated in parentheses; total number *N* = 65).

Certain aspects of the topic of vaccination are considered particularly interesting by the students (see Figure 2).

Approximately one-third (34%) of the statements related to interest in the topic of vaccination are connected to how vaccines work in the body. The primary reasons cited include the relevance to personal health and the connection to one’s own body.

Seventeen percent of the statements referred to student interest in vaccine development and research, with the COVID-19 pandemic and the topic’s high relevance to everyday life being the main factors driving this interest.

Social and ethical aspects accounted for a quarter (25%) of the expressions of interest, particularly in relation to misinformation about vaccination and vaccine hesitancy. Students expressed a desire to form their own opinions through better knowledge of vaccination, allowing them to more effectively asses and evaluate misinformation.

The topic of disease symptoms was also frequently mentioned, with 15% of the statements reflecting interest in this area. Experts confirm that students find this aspect especially engaging when symptoms are discussed in detail and supported by visual aids such as images.

Finally, some statements (9%) addressed special functions of the immune system and vaccinations in general.

### 4.2 Pathogen-specific interest

The students were asked to express their interest in three specific pathogens (Coronavirus, HPV, and Measles virus) and rank them. The Coronavirus and the topic of the COVID vaccine were rated as the most interesting by all respondents. HPV ranked second, while the Measles virus was rated as interesting by only a quarter of the students.

The reasons for this were particularly the connection to their own bodies and daily life, societal relevance, and their own knowledge—depending on how strongly these factors were present (or not), they either fostered or hindered interest.

#### 4.2.1 COVID-19

Nearly all of the students surveyed indicated a strong interest in the topic of COVID-19. Only one student stated that he/she did not want to learn anything about COVID-19, reasoning that the topic had been repeated and discussed so often that there was hardly anything new to learn about it.

Statements indicating student interest in the topic of COVID-19 and the COVID vaccine were most often associated with relevance to daily life and topicality. The students also mentioned finding the topic interesting because, although they already had some prior knowledge, there were still many aspects they only knew about superficially and would like to learn more about.

The strong interest in learning more about COVID-19 and the COVID vaccine was also confirmed by the teachers.

In connection with COVID, the topic of vaccine development and research was mentioned particularly frequently.

#### 4.2.2 HPV

Regarding the topic of HPV, students reported having little to no knowledge about the consequences of an HPV infection or the vaccine. This observation was corroborated by the teachers, who confirmed that students’ prior knowledge about HPV is limited and often restricted to its association with cervical cancer.

The students mentioned that they all received the vaccination because “that’s just what you do” and because it is recommended. While they frequently observed their peers getting vaccinated against HPV, they did not engage in further discussions about it.

About half of the students expressed interest in HPV, citing personal relevance as their primary reason. The other half initially showed indifference to the topic. However, after reading the fact sheets containing additional information about HPV, the number of students interested in the subject increased.

#### 4.2.3 Measles

Twenty-five percent of all students interviewed expressed an interest in the topic of measles. They mentioned that while they understood the dangers of contracting the measles virus and acknowledged having been vaccinated against it long ago, they had little knowledge about the disease itself. The lack of interest in measles was primarily attributed to its perceived low personal relevance and impact on their lives.

#### 4.2.4 Other pathogens and diseases

Students also expressed a desire to learn more about exotic diseases that require vaccination, particularly those encountered when traveling abroad. One of the teachers interviewed confirmed that her students found the topic of exotic diseases and vaccinations very engaging in class, mainly due to the focus on the symptoms associated with these the diseases.

### 4.3 Designing vaccine education

In the interviews with students, teachers, and experts, insights were shared on how to design lessons that foster interest, including both general methodological-didactic advice and specific suggestions for teaching about vaccination. The experiences and perspectives of students and educators on engaging with experts were also explored, along with the challenges and opportunities of organizing vaccination-related lessons within the school environment.

#### 4.3.1 Designing interest-promoting biology classes in general

Students find their biology classes more motivating when they have opportunities to work in groups and exchange ideas with one another, particularly through group work and station learning. They cited not only enjoyment but also a sense of control over their own learning process and the opportunity for differentiated learning as key reasons for this preference.

Additionally, students feel more competent when the subject matter is well-illustrated and they have ample time to engage with it. They expressed a strong desire to help shape the learning process, including the choice of content covered in class and the methods used to explore it.

Furthermore, students indicated that lessons become more interesting when they are relevant to everyday life, as it helps them see the personal significance of the content.

#### 4.3.2 Designing interest-promoting lessons on vaccination

The topic of immunobiology and vaccination is generally perceived as complex, making the methodological-didactic approach and effective visualization particularly important, according to the interviewees.

Students find case studies especially engaging, as they help illustrate the subject matter and create personal connections to the topic. They also reported feeling more competent when they have opportunities to apply their knowledge through transfer tasks, allowing them to explore additional aspects of the content independently.

Creative and playful approaches, such as collaborative activities and visual aids, can help simplify the complexity of the topic, fostering a more engaging and interest-driven lesson.

Give the social relevance and controversies surrounding vaccination, respondents stressed the importance of approaching the subject with factual accuracy and sensitivity. Students noted that it is crucial to ensure no one is marginalized or excluded for their views, whether by teachers or peers, when discussing the topic in class. Teachers highlighted the need to equip students with evaluation skills to help them make informed, fact-based decisions.

However, some students expressed reservations about addressing social and ethical aspects in biology class. While they acknowledged the importance of the topic, they felt that ethical debates and discussions of social issues would be better suited to social studies classes, arguing that biology should focus primarily on scientific content.

#### 4.3.3 Opening schools: meetings and interaction with experts

The interviews also explored how interactions with experts on the topic of vaccination can foster student interest in the classroom.

Students noted that while expert presentations often contain valuable biological content, they can sometimes be too complex and not methodically engaging. The frequent use of specialized language was identified a barrier to understanding, leaving students struggling to grasp the content.

Some students also shared experiences where they felt not taken seriously by the experts, which negatively affected their interest and engagement. Conversely, expert interactions were perceived positively and as more engaging when the experts adopted an appropriate approach, making the topics tangible and accessible. This allowed even complex or unfamiliar to become understandable. Students valued these interactions with experts because they offered new perspectives and authentic insights into the subject matter.

Students expressed a preference for smaller, more intimate settings for interacting with experts, rather than larger public events. Younger experts, such as medical students, were perceived as more relatable and easier to communicate with, possibly due to the smaller age gap.

In terms of content, students were especially interested in learning about the experts’ research and processes. From the experts’ point of view, older students in particular showed an interest in careers and educational paths related to the topics discussed.

#### 4.3.4 School organizational challenges

Teachers noted that designing lessons on vaccination is time-consuming and requires significant organizational effort, primarily because the topic is not deeply embedded in the core curriculum. This limitation prevents the extensive coverage of the topic that would be necessary for students to grasp its complexity and importance.

The preparation for such a lesson requires more time than other topics due to the lack of readily available materials from standard educational publishers, forcing teachers to search for or create content themselves.

Additionally, making the topic accessible and relevant, such as by inviting experts or organizing visits to laboratories and research facilities, involves considerable logistical efforts and coordination with colleagues. Often, there is also a lack of contacts with suitable experts who are willing to engage with students.

## 5 Discussion

### 5.1 Vaccination in school curricula

Our findings indicate that the omission of vaccination as a focused topic within school curricula results in it being addressed only superficially during instruction, leading to correspondingly low levels of knowledge among students. Although both teachers and students consider the topic important, it currently plays only a minor role in classroom instruction within the sample studied. The surveyed teachers report that they make efforts to address the topic with their students; however, they often reach their limits due to the lack of allocated time in the curriculum and the shortage of teaching-specific materials, particularly when they aim to integrate external experts into their lessons. The involvement of experts is also occasionally hampered by the lack of relevant contacts.

To ensure the adequate coverage of vaccination in the classroom, it would be highly beneficial to more thoroughly integrate the topic into the curriculum. This would provide teachers with the necessary time to address the complexities of the subject in greater depth and also facilitate the involvement of medical experts or organize visits to laboratories and medical facilities for students. Additionally, textbook publishers would then also be more likely to develop and provide appropriate learning materials for the topic.

### 5.2 Students’ interest in vaccination

As shown in other studies [e.g., (8, 26, 41)], students are particularly interested in human biology topics during class. Our data suggest that in the case of infectious diseases and vaccination this can be explained by the personal relevance of the subject matter and its connection to their own health. Additionally, human biology topics are perceived as tangible, which facilitates the understanding of subject content.

The topic of vaccination is generally considered interesting by students, a view that is also confirmed by teachers and experts. Students value being informed about diseases, vaccinations, and their mechanisms of action to make informed decisions. Certain aspects of the vaccination topic are particularly interesting to students: They are especially intrigued by how vaccines work in the body and by research related to vaccines. Additionally, disease symptoms are of interest, particularly when visualized or when students can relate personally to the diseases. Ethical and social aspects of vaccination are also seen as engaging by students, especially issues like vaccine hesitancy and misinformation. Students express a desire to recognize such information and form their own opinions.

There are noticeable differences in interest concerning different diseases and their corresponding vaccines, which are linked to the topic’s relevance, personal impact, symptoms, and individual agency. The students surveyed find COVID-19 more interesting than HPV and measles because COVID-19 is current and highly relevant to their lives. The cognitive component of interest is particularly strong here, as students want to understand the scientific information in order to make personal decisions about whether to get vaccinated. HPV is perceived as interesting specifically by adolescents because the vaccinations are given at this age. Despite the high personal relevance, decision-making is not the primary concern for students in relation to HPV. In the context of those surveyed, the HPV vaccination is “routinely” administered and not questioned further. As a social norm, the vaccination is considered “normal,” and there is a reliance on parents and doctors. This is also reflected in very low levels of knowledge about HPV. The students surveyed find it interesting to learn that HPV can cause various types of cancers and is also relevant to boys. The novelty factor plays a role here in fostering interest. Measles is perceived as the least interesting by the respondents because vaccinations occur in early childhood. Thus, they happened long ago, and students have no influence or decision-making power regarding them, a situation further reinforced by mandatory vaccination. Consequently, personal relevance in this case is very low. A certain level of interest is sparked when students recognize the societal relevance—for example, in the context of herd immunity—or learn about measles symptoms that surprise them (novelty). Symptoms and their severity also make other diseases and corresponding vaccinations interesting, such as tropical diseases, to which there is otherwise little personal connection—except in the case of travel.

### 5.3 Designing interest-promoting vaccine education in school

While there is a lack of directly comparable studies on this specific topic, the interview data confirm findings related to interest-promoting education in other contexts, which recommend student-centered and activating approaches [e.g., (22, 42)]. The students’ statements reveal interest-promoting factors aligned with the *basic needs*, whose fulfillment contributes to the development of interest—i.e., the need for experiencing competence, social relatedness, and autonomy (16, 18). For example, students find group work particularly engaging because it allows them to exchange ideas with peers and actively engage with the content on their own. They appreciate being able to independently and responsibly acquire content at their own pace, as is the case with station learning. Playful and creative approaches are also highly valued, especially when they promote group learning and help reduce the complexity of the topic through visualizations and illustrations. Another important point mentioned by the students in the interviews is the relevance to everyday life, which should be evident to them, as recognizing the significance of the topic for themselves positively influences their interest. Additionally, they want to be involved in lesson planning or see value in teachers considering their suggestions and ideas in lesson design. These findings align with existing educational theories and can inform pedagogical practices in other schools and other national contexts, especially those with similar curricula and age groups.

Specifically regarding the topic of vaccination, the students’ areas of interest should be taken into account and deliberately incorporated into lesson planning. This is especially important given that vaccination is a very complex subject, requiring an in-depth engagement with the content. This has been confirmed once again by the present data.

The interest and engagement with the topic are enhanced when students perceive a connection to their own lives and personal experiences, thereby recognizing the topic as relevant and important. This can be achieved in the classroom by embedding the subject matter in illustrative and authentic contexts, such as through the use of personal stories and case studies. The students expressed a desire for such concrete examples, as these make it easier for them to relate to the content. The use of (historical) case studies is also recommended by the experts interviewed and aligns with the recommendations of Reiss (4). When students learn how dangerous an infection with a particular pathogen can be, or when they can view the consequences of the disease through photos or videos, their interest is heightened. Therefore, it is advisable to specifically address the diseases and their symptoms when discussing the topic of vaccination and to demonstrate how vaccinations can help mitigate or even prevent these symptoms.

Additionally, meeting with experts can significantly foster students’ interest, enhancing authenticity, practical relevance, and clarity. However, it is crucial that communication is appropriate for the audience and that the students’ wishes and interests are taken into account. Moreover, students need to feel taken seriously; otherwise, their needs for a feeling of competence and social relatedness (basic needs) may not be met, which could negatively affect the development of their interest or willingness to engage with the topic of vaccination.

The interviewed students emphasize the importance of creating a respectful and trusting atmosphere when teaching about vaccination and addressing social and ethical questions, which aligns with the recommendations formulated by Reiss (9). This is crucial to prevent bullying or exclusion if some students hold different views from their peers. A factual approach is recommended in such cases, as students have found it to be particularly helpful based on their own experiences. Involving experts who are not part of the class’s social dynamics and can provide practical, evidence-based information may be helpful in ensuring that the topic of vaccination is addressed in a well-informed and objective manner.

Meeting with experts can be particularly effective in fostering interest when direct contact and exchange are facilitated. In small group settings, especially with younger experts where students may feel less apprehensive, students tend to be more open and take the opportunity to ask questions. These questions may not only pertain to work processes and research but also to career paths, thereby promoting not only vaccine education but also career orientation. A positively perceived interaction between experts and learners that positively impacts interest development requires careful planning and close collaboration between the teacher and the experts. The teacher acts as a link between the students and the experts and should be in contact with the experts beforehand to share the students’ knowledge level and areas of interest. When the content and teaching methods are determined jointly with the experts, it helps ensure that students do not feel overwhelmed during the meeting and that the content aligns with their interests.

Since the topic of vaccination is both highly relevant to society and the subject of controversial debate, it is important to adequately prepare students. In addition to a solid understanding of immunobiology, they need evaluation skills to make informed and fact-based decisions. Both teachers and students alike express the need for and emphasize the importance of these skills. However, there is disagreement among respondents about when and where ethical and social aspects should be addressed in school. Some students do not see a place for this in biology class and would prefer to engage with these topics in social science subjects. This is surprising because argumentation and decision-making is explicitly included in the biology curriculum in Germany, where it is clearly stated that evaluative competencies are part of subject competence and must therefore be appropriately taught. Such competencies are “demonstrated by their (the students’) understanding of both subject-specific and interdisciplinary perspectives and evaluation methods, as well as by their ability to use these to assess statements or data, based on various criteria. These competences also include the ability to form well-founded opinions, make decisions based on ethical considerations, and reflect on decision-making processes and their consequences” [(43), p. 10]. Our data suggest that this area, traditionally not embedded in the science curricula, is still not comprehensively addressed in current teaching practices.

### 5.4 Methods discussion

In selecting interview participants, special attention was given to including students from two different age groups. This approach was intended to provide comprehensive coverage of the school grades in which the topic of vaccination is typically addressed within the educational context. However, it is important to acknowledge that the study’s findings are based on a relatively small sample size, which may limit the breath of perspectives. Although the purposive sampling ensured rich information, the findings cannot be generalized to all students and may therefore not fully represent students in other educational systems or cultural backgrounds. Moreover, all participants held a generally positive attitude toward vaccination. While this could introduce a potential bias, it is worth noting that the attitude of the participants plays only a secondary role concerning the research question at hand.

It is also essential to consider the inherent subjectivity in both the collection and analysis of qualitative data. The responses of interviewees can be influenced by various factors, such as the atmosphere during the conversation. This atmosphere is, in turn, shaped by the relationship between the interviewer and the interviewees. For instance, if the interviewees perceive the interviewer as likable and trustworthy, they are more likely to respond truthfully and maintain the flow of conversation (36).

Furthermore, while the analysis of the transcribed interview data was carried out using a thoroughly developed coding system, the categorization of responses can never be entirely free from subjective interpretation. Despite efforts to minimize bias through the structured use of codes, the assignment of statements to specific categories is inevitably influenced by the researchers’ perspectives. To enhance transparency in this process, numerous original quotes from the interviews, along with their corresponding categorizations and interpretations, have been included in the results section. This approach allows readers to critically assess how the data were interpreted and ensures that the connection between the raw data and the derived conclusions is clear and traceable.

## 6 Conclusion

This study highlights the crucial role of incorporating students’ perspectives into the design of vaccine education programs. Interviews with secondary school students, educators, and vaccination experts reveal that student engagement in immunobiology and vaccination topics significantly improves when educational content aligns with their interests, concerns, and prior knowledge.

Our findings indicate that students often come to the subject with limited background knowledge, leading to potential misunderstandings and disengagement. Tailoring educational approaches to address students’ interests and experiences not only enhances their engagement but also improves retention of information. This underscores the need for educational strategies that do more than deliver factual content—they must connect with students on a personal level to make the topic of vaccination more relevant and compelling.

To further engage students, it is essential for educators to involve them in developing educational materials and approaches. By actively listening to and addressing students’ concerns and questions, educators can create a more inclusive and effective learning environment. This approach not only educates students about the importance of vaccinations but also equips them with critical thinking skills for making informed decisions.

In conclusion, educators should consider integrating interactive and student-centered learning activities to enhance the students’ interest and engagement with the topic. This includes the use of group work, which promotes peer-to-peer interaction and active learning, as well as station learning that allows students to explore content at their own pace. Incorporating playful and creative approaches, such as quizzes or creating illustrations, can engage the students and help simplify complex topics such as immunobiology and vaccines. Additionally, integrating real-world examples in the lessons and providing illustrative contexts, such as personal stories or historical case studies, is essential, as it helps students see the relevance of the material to their own lives, fostering a deeper connection to the subject matter. Expert involvement, especially through direct interaction in small groups, enhances interest and can also contribute to career orientation. Educators are encouraged to collaborate closely with experts to tailor the content and teaching approach to the students’ knowledge level and interests. Lastly, to prepare students for societal debates, such as those surrounding vaccination, it is crucial to equip them with both a solid understanding of the scientific concepts and the evaluation skills needed to make informed decisions.

**Recommendations for educators**

1. *Incorporate interactive and student-centered learning*: Engage students by integrating interactive, student-centered activities into lessons on vaccination and immunobiology. Utilize group work to promote peer-to-peer interaction and active learning, and consider station learning to allow students to explore content at their own pace.
2. *Utilize playful and creative approaches*: Simplify complex topics by incorporating playful, creative methods such as quizzes, role-playing, or the creation of illustrations and visual aids. These strategies enhance engagement and make challenging material more accessible.
3. *Connect lessons to real-world contexts*: Use real-world examples, such as personal stories, or historical case studies, to make the subject matter more relevant to students’ lives. This connection fosters deeper understanding and interest in the topic.
4. *Involve experts for enhanced engagement*: Involve medical experts or public health professionals in teaching the topic of vaccination to provide real-world insights. Small group meetings with experts can not only deepen student interest but also contribute to career exploration.
5. *Collaborate with experts to tailor content*: Work closely with external experts to ensure that lesson content is adapted to the students’ knowledge level and interests. This collaboration can enhance both the quality and relevance of the educational experience.
6. *Equip students for societal debates*: Prepare students to engage in discussions about vaccination by teaching them both the scientific facts and critical evaluation skills necessary to assess information and make informed decisions.

The insights gained from this study will contribute to the ongoing development of educational strategies and initiatives, such as the international open schooling project “Multipliers,” aimed at improving science education and public health outcomes. By focusing on student-centered communication and the collaboration between schools and medical professionals, we can enhance the effectiveness of vaccine education and ultimately support the broader goal of increasing vaccination rates and public health awareness.

## Data Availability

The raw data supporting the conclusions of this article will be made available by the authors, without undue reservation.

## Footnotes

^1^https://multipliers-project.org/

## References

[1] World Health Organization. Ten threats to global health in 2019. (2019). Available at: https://www.who.int/news-room/spotlight/ten-threats-to-global-health-in-2019

[2] Högemann A Kramer H Mais A Reinecke K Speer R. Ärztliche Gesundheitsbildung in Schulen – ein wichtiger Beitrag zur Steigerung der HPV-Impfmotivation. Epidemiologisches Bull. (2022) 36:11–22. doi: 10.25646/10463

[3] Schott E Schaller K Mons U Ouédraogo N. Ansätze zur Steigerung der HPV-Impfquote in Deutschland – Hindernisse und Chancen: Eine qualitative Studie. ZEFQ J. (2022) 170:29–37. doi: 10.1016/j.zefq.2022.02.002 35490121

[4] Reiss MJ. Trust, science education and vaccines. Sci & Educ. (2022) 31:1263–80. doi: 10.1007/s11191-022-00339-x, PMID: 35497258 PMC9039980

[5] Budke A Meyer M. Fachlich argumentieren lernen: Die Bedeutung der Argumentation in den unterschiedlichen Schulfächern In: Budke A Kuckuck M Meyer M Schäbitz F Schlüter K Weiss G, editors. Fachlich argumentieren lernen. Didaktische Forschungen zur Argumentation in den Unterrichtsfächern. Münster; New York: Waxmann (2015).

[6] Krapp A. Interesse, Lernen und Leistung. Neue Forschungsansätze in der Pädagogischen Psychologie. Zeitschrift für Pädagogik. (1992) 38:747–70.

[7] Schiefele H. Interesse – Neue Antworten auf ein altes Problem. Zeitschrift für Pädagogik. (1986) 32:153–62.

[8] Baram-Tsabari A Sethi RJ Bry L Yarden A. Identifying students' interests in biology using a decade of self-generated questions. Eurasia J Math Sci Technol Educ. (2010) 6:63–75. doi: 10.12973/ejmste/75228

[9] Reiss MJ. Evolution education: treating evolution as a sensitive rather than a controversial issue. Ethics Educ. (2019) 14:351–66. doi: 10.1080/17449642.2019.1617391

[10] Renninger KA Hidi SE. The power of interest for motivation and engagement. New York: Routledge/Taylor & Francis Group (2016).

[11] Renninger KA Hidi SE. To level the playing field, develop interest. Policy Insights Behav Brain Sci. (2020) 7:10–8. doi: 10.1177/2372732219864705

[12] Ainley M Hidi S Berndorff D. Interest, learning, and the psychological processes that mediate their relationship. J Educ Psychol. (2002) 94:545–61. doi: 10.1037/0022-0663.94.3.545

[13] Hidi S Renninger KA. The four-phase model of interest development. Educ Psychol. (2006) 41:111–27. doi: 10.1207/s15326985ep4102_4

[14] Krapp A Prenzel M. Research on interest in science: theories, methods, and findings. Int J Sci Educ. (2011) 33:27–50. doi: 10.1080/09500693.2010.518645

[15] Renninger KA Hidi S. Revisiting the conceptualization, measurement, and generation of interest. Educ Psychol. (2011) 46:168–84. doi: 10.1080/00461520.2011.587723

[16] Deci EL Ryan RM. Die Selbstbestimmungstheorie der Motivation und ihre Bedeutung für die Pädagogik. Zeitschrift für Pädagogik. (1993) 39:223–38.

[17] Großmann N Wilde M. Promoting interest by supporting learner autonomy: the effects of teaching behaviour in biology lessons. Res Sci Educ. (2020) 50:1763–88. doi: 10.1007/s11165-018-9752-5

[18] Krapp A. Basic needs and the development of interest and intrinsic motivational orientations. Learn Instr. (2005) 15:381–95. doi: 10.1016/j.learninstruc.2005.07.007

[19] Wilde M. Retzlaff-Fürst C. Scheersoi A. Basten M. Groß J. (2019). Non-formales Biologielernen mit Schulbezug. Biologiedidaktische Forschung: Erträge für die Praxis. 251–268.

[20] Dohn NB. Upper secondary students’ situational interest: a case study of the role of a zoo visit in a biology class. Int J Sci Educ. (2013) 35:2732–51. doi: 10.1080/09500693.2011.628712

[21] Harackiewicz JM Knogler M. Interest: theory and application In: Elliot AJ Dweck CS Yeager DS, editors. Handbook of competence and motivation: Theory and application. New York: Guilford Publications (2017). 334–52.

[22] Hasni A Potvin P. Student’s interest in science and technology and its relationships with teaching methods, family context and self-efficacy. Int J Environ Sci Educ. (2015) 10:337–66. doi: 10.12973/ijese.2015.249a

[23] Holstermann N Grube D Bögeholz S. Hands-on activities and their influence on students´ interest. Res Sci Educ. (2010) 40:743–57. doi: 10.1007/s11165-009-9142-0

[24] Potvin P Hasni A. Interest, motivation and attitude towards science and technology at K-12 levels: a systematic review of 12 years of educational research. Stud Sci Educ. (2014) 50:85–129. doi: 10.1080/03057267.2014.881626

[25] Swarat S Ortony A Revelle W. Activity matters: understanding student interest in school science. J Res Sci Teach. (2012) 49:515–37. doi: 10.1002/tea.21010

[26] Elster D. Student interests – the German and Austrian ROSE survey. J Biol Educ. (2007) 42:5–10. doi: 10.1080/00219266.2007.9656100

[27] Holstermann N Bögeholz S. Interesse von Jungen und Mädchen an naturwissenschaftlichen Themen am Ende der Sekundarstufe I. Zeitschrift für Didaktik der Naturwissenschaften. (2007) 13:71–86. doi: 10.25656/01:31607

[28] Tsai Y-M Kunter M Lüdtke O Trautwein U Ryan MR. What makes a lesson interesting? The role of situational and individual factors in three school subjects. J Educ Psychol. (2008) 100:460–72. doi: 10.1037/0022-0663.100.2.460

[29] Schlopsna M. man hört immer nur so mRNA, aber was ist das eigentlich? – Eine empirische Untersuchung zum Interesse der Schüler*innen am Thema Impfen im Biologieunterricht. Master’s thesis. Bonn (GER): University of Bonn (2023).

[30] Dillon J Avraamidou L. Towards a viable response to COVID-19 from the science education community. J Activ Sci Technol Educ. (2020) 11:1–6. doi: 10.33137/jaste.v11i2.34531

[31] Long DE. The politics of teaching evolution, science education standards, and being a creationist. J Res Sci Teach. (2012) 49:122–39. doi: 10.1002/tea.20445

[32] Lederman NG. Nature of science: past, present, and future In: Abell SK Lederman NG, editors. Handbook of research on science education. Mahwah, NJ: Lawrence Erlbaum Associates (2007). 831–79.

[33] McComas W. Nature of science in science instruction: Rationales and strategies. Cham: Springer Nature (2020).

[34] Martínez-Pena I Puig B Uskola A. One health education for criticality on vaccination in teacher training. Front Public Health. (2024) 12:1408965. doi: 10.3389/fpubh.2024.1408965, PMID: 39131576 PMC11312376

[35] Bortz J Döring N. Forschungsmethoden und Evaluation für Human- und Sozialwissenschaftler, 4. überarbeitete Auflage ed. Heidelberg: Springer (2006).

[36] Niebert K Gropengießer H. Leitfadengestützte interviews In: Krüger D Parchmann I Schecker H, editors. Methoden in der naturwissenschaftsdidaktischen Forschung. Berlin, Heidelberg: Springer (2014). 121–32.

[37] Miles MB Huberman AM. Qualitative data analysis: an expanded sourcebook. Thousand Oakes, London, New Delhi: Sage Publications (1994).

[38] Dresing T Pehl T. Praxisbuch Interview, Transkription & Analyse. Anleitungen und Regelsysteme für qualitativ Forschende. 8. Auflage ed. Marburg: Eigenverlag (2013).

[39] Krüger D Riemeier T. Die qualitative Inhaltsanalyse – eine Methode zur Auswertung von Interviews In: Krüger D Parchmann I Schecker H, editors. Methoden in der naturwissenschaftsdidaktischen Forschung. Berlin, Heidelberg: Springer (2014). 133–45.

[40] Kuckartz U. Qualitative Inhaltsanalyse. Methoden, Praxis, Computerunterstützung. 4th ed. Weinheim, Basel: Beltz Juventa (2018).

[41] Uitto A. Interest, attitudes and self-efficacy beliefs explaining upper-secondary school students’ orientation towards biology-related careers. Int J Sci Math Educ. (2014) 12:1425–44. doi: 10.1007/s10763-014-9516-2

[42] Scheersoi A. Bögeholz S. Hammann M. (2019). Biologiedidaktische Interessenforschung: Empirische Befunde und Ansatzpunkte für die Praxis. Biologiedidaktische Forschung: Erträge für die Praxis, 37–55.

[43] Kultusminister Konferenz (2020). Bildungsstandards im Fach Biologie für die Allgemeine Hochschulreife. Beschluss der Kultusmunisterkonferenz vom. Available at: https://www.kmk.org/fileadmin/Dateien/veroeffentlichungen_beschluesse/2020/2020_06_18-BildungsstandardsAHR_Biologie.pdf (Acessed August 23, 2023).

